# Evaluation and Optimization of Antibiotics Resistance Profile against *Clostridium perfringens* from Buffalo and Cattle in Pakistan

**DOI:** 10.3390/antibiotics10010059

**Published:** 2021-01-08

**Authors:** Muhammad Umar Zafar Khan, Muhammad Humza, Shunli Yang, Muhammad Zahid Iqbal, Xiao Xu, Jianping Cai

**Affiliations:** 1 State Key Laboratory of Veterinary Etiological Biology of Veterinary Parasitology of Gansu Province, Lanzhou Veterinary Research Institute, Chinese Academy of Agricultural Sciences, Lanzhou 730046, China; umarzafar8@caas.cn (M.U.Z.K.); yangshunli@caas.cn (S.Y.); xu05xiao@163.com (X.X.); 2 Jiangsu Co-Innovation Center for the Prevention and Control of Important Animal Infectious Disease and Zoonoses, Yangzhou University, Yangzhou 225009, China; 3 Key Laboratory of Agro-products Quality and Safety Control in Storage and Transport Process, Ministry of Agriculture and Rural Affairs/Institute of Food Science and Technology, Chinese Academy of Agricultural Sciences, Beijing 100193, China; muhammadhumza1992@outlook.com; 4 Department of Plant Pathology, University of Agriculture, Faisalabad 38000, Pakistan; 5 Department of Veterinary Medicine, University of Veterinary and Animal Sciences, Outfall Road, Lahore 54000, Pakistan; zahid.iqbal@uvas.edu.pk

**Keywords:** *Clostridium perfringens*, ciprofloxacin, ampicillin, cefotaxime, optimization, response surface methodology

## Abstract

*Clostridium perfringens* is a serious threat to successful bovine farming. It causes severe damage to the buffalo and cattle health causing a drastic reduction in milk and meat production. In Pakistan, *C. perfringens* is a constant threat, and for its management, antibiotics are mostly used. Most bovine farmers use a single antibiotic to suppress the bacterial infection which in turn, increases the antimicrobial resistance (AMR) against the particular antibiotic. To reduce the resistance, the administration of multiple antibiotics in their standard doses at different times can be a possible remedy to manage the AMR and reduce their viability. This study aims to evaluate the effect of 11 commonly used antibiotics at their standard concentrations for inhibiting 33 strains of *C. perfringens* from five districts of Punjab province in Pakistan. Based on the zone of inhibition, ciprofloxacin, ampicillin, and cefotaxime (CAC) at their standard concentrations effectively inhibited the bacterium. These antibiotics showed appropriate significance statistically, i.e., correlation, Chi-square test, and cluster analysis. Optimization of these antibiotics using response surface methodology (RSM) revealed that the selected antibiotics from medium to high range not only reduce the bacterial propagation but also their population up to a considerable extent. Hence, the health of milk- and meat-producing large animals could be improved, which will be cost-effective and less harmful to the animal, human health, and the environment. Moreover, optimized administration of the selected antibiotics would reduce the impact of drug-resistant superbugs.

## 1. Introduction

Pakistan is an agriculture-based country, and its livestock industry plays a pivotal role in the economy, contributing 11.7% to the total gross domestic product (GDP) during the financial year 2019–2020. Currently, there are 90.8 million herds of buffalo and cattle in Pakistan, sharing 96.87% and 48.91% of the total milk and beef production, respectively [1]. This successful production of milk and meat is under threat of certain fatal pathogens in which *Clostridium perfringens* is the most prominent one. *C. perfringens* is an anaerobic, Gram-positive, rod-shaped bacterium, [2,3] which produces over 20 different toxins including enterotoxin (CPE), beta-2 (CPB2), epsilon (ETX), theta/perfringolysin O (PFO), TpeL, BecA/B, Nan (I, J), Net (B, E, F, G), lambda, clostripain, delta, iota (CPI), kappa, mu, alpha (CPA), and beta (CPB) toxin [4,5].

Several pathogenic microorganisms and their resistomes have circulated in our environment. These resistomes are mainly evolved from hospitals, pharmaceutical companies, and livestock producers’ unmonitored waste that contains antimicrobial substances that have been circulating in our environment. Among all pathogens, *Staphylococcus* spp., *Campylobacter* spp., *Enterococcus* spp., *Salmonella* spp., and Extended Spectrum Beta-Lactamase (ESBL) producing Enterobacteriaceae are most commonly found in the environment [6,7].

For decades, antibiotics are not only used to prevent and treat a variety of human and animal diseases but also used as growth promoters in livestock [8]. Antibiotics inhibit pathogenic microorganisms and pathogens fight back and find new ways to survive through resistance mechanisms, requiring the discovery of new antibiotics. The antibiotic resistance in pathogens is rapidly increasing, while the rate of discovery and production of new antibiotics are very slow and tedious [9,10]. Hence, time is needed to develop new strategies for the production and optimization of new antibacterial agents. Antibiotics have also been a part of chemometric analysis as they have been tested in various fields such as veterinary sciences and wastewater analysis which confirms the availability and separation of antibiotics up to an appropriate level [11] 

The objective of this study is to highlight common antibiotics in veterinary practices and to monitor a blind approach towards managing threat of emerging resistance in bovines. For this, we used statistical analysis and models to estimate the prudent use of antibiotics in the areas having more livestock farming. Although very limited data are available on AMR modeling, a model could be developed to forecast the emergence of pathogen resistance. Considering the optimization under a statistical perspective, there is an analytical optimization technique which is called response surface methodology [12]. It is a mathematical and statistical technique that involves fitting the experimental variables in a polynomial equation and determines the effect of each variable. It also depicts the trend of a data set to make statistical provisions. It is applied to a response or a set of responses from the variables of interest. The main objective of this analysis is to optimize the variables for better performance in a systematic way. The approach of multivariate design is helpful in the optimization of every single variable and its interactive effects among them, and these types of methods are advantageous as they have advanced statistical evaluations with less time consumption and appropriate optimization of variables along with their interactions. The most frequently used analytical method for optimization is chemometry, due to their advantages such as minimum chemical consumption by reducing the number of experiments, and minimum laboratory work [13]. Further, results could be analyzed through the Box–Behnken method which is an extension of factorial design. This experimental design is arranged in such a way that each point is localized in the center and middle of the edges of a cube and increases the design points as the number of polynomial coefficients [14]. The main disadvantage of chemometry is that it could not undergo the experiment in an extreme condition where the chances of unsatisfactory results are very high [15,16]. For our study of evaluating antibiotics against *C. perfringens* strains, the method of optimization could be a good extrapolative strategy to foresee the antibiotic resistance among the strains.

## 2. Results

### 2.1. Isolation and Identification of C. perfringens from Buffalo and Cattle

All 33 isolates were detected as *C. perfringens* through biochemical tests, i.e., glucose (+), maltose (+), H_2_S reduction test (−), nitrate reduction test (+), gelatin liquefaction (+), and saccharose test (+). Afterward, 16S rRNA gene amplification was performed by PCR followed by sequences analysis. The representative sequences can be accessed at NCBI with accession number MT158886-MT158897.

### 2.2. Impact of Antibiotics against Clostridium perfringens Isolated from Buffalo and Cattle

Eleven antibiotics were tested against 33 isolates of *C. perfringens* based on inhibition zones produced by the bacterium in response to a particular antibiotic as shown in Table 1. Among them, ciprofloxacin was the best one showing inhibition of maximum strains, while amikacin and lincomycin showed no inhibition of all bacterial strains tested.

As mentioned in Table 1, out of a total 33 strains, 28 (85%) strains (15 from buffalo and 13 from cattle) were found to be highly susceptible (+++) and five (15%) strains were found to be moderately susceptible (++) against ciprofloxacin.

In response to ampicillin, 18 (55%) strains (12 from buffalo and six from cattle) were found to be highly susceptible (+++), 15 (45%) strains (seven from buffalo and eight from cattle) were found to be moderately susceptible (++).

Observing the effect of cefotaxime, 10 (30%) strains (seven from buffalo and three from cattle) showed highly susceptible (+++) response, 21 (64%) strains (10 from buffalo and 11 from cattle) exhibited moderately susceptible response (++), and two (6%) strains showed less susceptible (+) response.

In the case of penicillin, eight strains (24%) (five from buffalo and three from cattle) showed highly susceptible (+++) response and 25 (76%) strains (13 from buffalo and 12 from cattle) showed moderately susceptible (++) response.

In response to metronidazole, eight (24%) strains (four from buffalo and four from cattle) showed highly susceptible (+++) response and 25 (76%) strains (14 from buffalo and 11 from cattle) showed moderately susceptible (++) response.

Observing the effect of oxytetracycline, one (3%) strain showed moderately susceptible (++) response, 19 (58%) strains showed less susceptible (+) response, and 13 (39%) strains were found to be resistant (−) to oxytetracycline in both buffalo and cattle.

In response to tetracycline, three (9%) strains of the total (33) strains in both buffalo and cattle showed moderately susceptible (++) response, 16 (49%) strains showed less susceptible (+) response, and 14 (42%) strains exhibited resistant (−) behavior towards tetracycline.

Around 30 strains (90%) of the total strains in both buffalo and cattle displayed less-susceptible (+) response, while three (10%) strains showed resistance (−) towards erythromycin.

A total of five (15) strains (two in buffalo, three in cattle) showed moderately susceptible (++) response and 28 (85%) strains (16 in buffalo, 12 in cattle) exhibited less susceptible (+) response to Vancomycin.

Lincomycin and amikacin showed no inhibitory response against all the strains, and all the strains were found to be resistant (−) to both antibiotics. The antibiotics which were tested against *C. perfringens* strains were also subjected to a Chi-square test which concluded that ciprofloxacin, ampicillin, and cefotaxime showed a maximum degree of inhibition to all strains, while other antibiotics were subsequently less inhibitive against all the strains. Lincomycin and amikacin showed no inhibition (Table 2).

**Figure 1:**
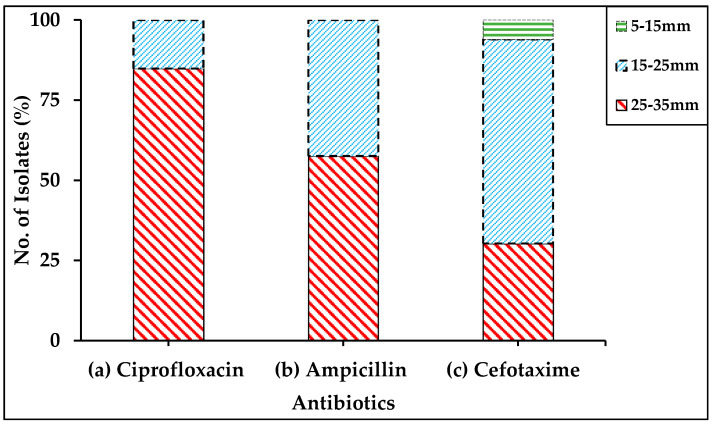
Stacked bar graph showing efficacy of three antibiotics (a) ciprofloxacin, (b) ampicillin, and (c) cefotaxime against the 33 strains of *C. perfringens* at their standard concentrations based on zone of inhibition in buffalo and cattle of Punjab province in Pakistan.

The effect of selected antibiotics showing a significant effect against *C. perfringens* was observed on a stacked bar graph (Figure 1). Ciprofloxacin showed a wide range of inhibition reactions against the various strains of *C. perfringens*, which confirmed that it is effective against most of the bacterial strains based on the inhibition zones, while ampicillin and cefotaxime showed less inhibition as compared to ciprofloxacin. From these stacked bar graphs, it can be suggested that the application of these antibiotics at different times may have a deeper impact on the activity of the bacterium. Furthermore, selected antibiotics have varied impacts on all strains, so this variation in activity among the antibiotics could be very helpful for reducing bacterial propagation.

### 2.3. Cluster Analysis of Antibiotics Used against Isolates of C. perfringens

**Figure 2:**
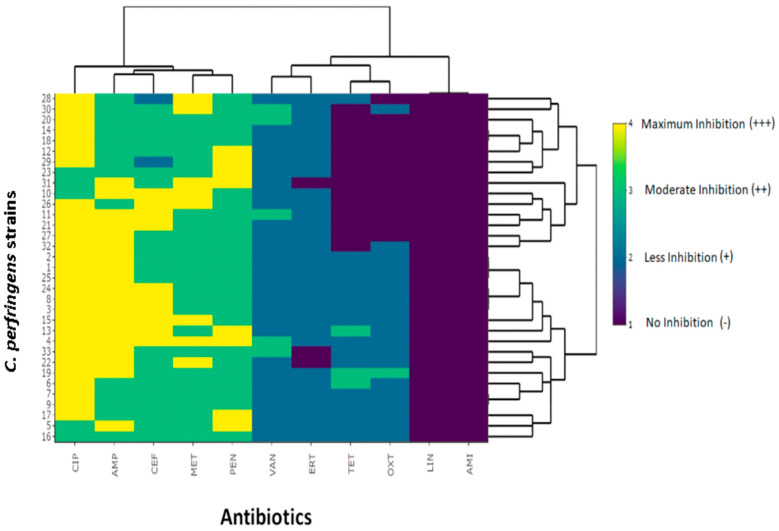
Cluster analysis of tested 11 antibiotics (CIP = Ciprofloxacin, AMP = Ampicillin, CEF = Cefotaxime, MET = Metronidazole, PEN = Penicillin, VAN = Vancomycin, ERT = Erythromycin, TET = Tetracycline, OXT = Oxytetracycline, Lincomycin (LIN), and Amikacin (AMK)) against *C. perfringens* isolates from Punjab province of Pakistan at their standard concentrations. The cluster analysis of all antibiotics determining the zone of inhibition is depicted in heat map form. The color scale depicts the extent of inhibition by antibiotics.

As shown in Figure 2, 9 out of 11 antibiotics showed inhibitory activity against the isolates. However, ciprofloxacin, ampicillin, and cefotaxime showed significant inhibition of bacterial growth. These antibiotics could potentially reduce the growth and severity of bacterial isolates. Ciprofloxacin showed maximum inhibition, while most of the antibiotics showed medium to slightly strong response against bacterial growth, which confirms that the antibiotics have broad-spectrum reactivity towards inhibition of bacterium.

### 2.4. Correlation of Antibiotics Used against Isolates of C. perfringens

**Figure 3:**
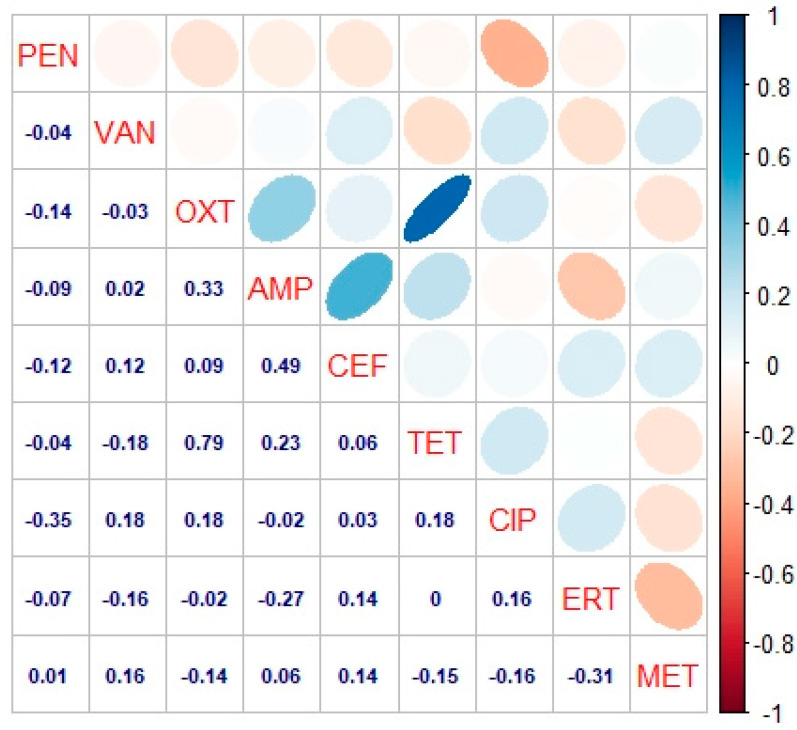
Correlation analysis among the antibiotics against *C. perfringens* isolates at their standard concentrations. The digits in blue depict and the ellipses describe the r-value (correlation value) of the tested antibiotics. PEN = Penicillin, VAN = Vancomycin, OXT = Oxytetracycline, AMP = Ampicillin, CEF = Cefotaxime, TET = Tetracycline, CIP = Ciprofloxacin, ERT = Erythromycin and MET = Metronidazole. Red and light red colors in oval and ellipses represent negative correlation, while blue and light colors in oval and ellipses represent positive correlation among the antibiotics as per scale mentioned on the left side of the matrix. As Linocmycin (LIN) and Amikacin (AMK) showed no inhibition zone, so their correlation was not observed; hence, these antibiotics were excluded from the matrix.

The correlation analysis results identified a significant correlation with inhibition. As shown in Figure 3, it was found that penicillin showed a negative correlation with all other antibiotics at their standard concentration except metronidazole in response to inhibition. This depicts that penicillin shows an antagonistic effect if applied in combination with other antibiotics, which reduces the inhibitory activity against the bacterium on a culture plate. Vancomycin and Oxytetracycline both were found to be negatively associated with bacterial growth. Ampicillin showed a positive and a significant correlation as it showed better antibacterial activity. Checking the activity of cefotaxime, the inhibitory activity was found to be less, and the bacterial isolates showed tolerance or less mortality against cefotaxime. Tetracycline had a less significant inhibitory effect and the correlation was positively significant. Ciprofloxacin showed a significantly positive correlation with inhibition as the inhibitory activity of the antibiotic was found to be maximum and effective against the bacterial isolates. Erythromycin showed a positive and significant correlation with inhibition of bacterial isolates as the inhibitory activity of the antibiotic was found to be minimum. Metronidazole had less inhibition against the bacterial isolates as the inhibitory performance of the antibiotic was negatively correlated but had a significant impact. Thus, correlation proves that selected antibiotics have significant performance in response to bacterial growth at a 5% level of significance (*p* ≤ 0.05).

### 2.5. Optimization of Post-Evaluation Selected Antibiotics against Bacterial Isolates Inhibition via Response Surface Methodology

For all isolates which were used in the study, the effect of antibiotics was assessed based on response surface methodology via the Box Behnken design [16]. The following quadratic response surface model was fitted to the data.

$$Y=\beta_{o}+\overset{3}{\underset{i=1}{\sum }}\beta_{\mathrm{ii}}F_{i}+\overset{3}{\underset{i=1}{\sum }}\beta_{\mathrm{ii}}F_{i}^{2}+\sum \overset{3}{\underset{i<j=1}{\sum }}\beta_{\mathrm{ij}}F_{i}F_{j}+\varepsilon$$

where “Y” is inhibition of bacterial isolates; “β_0_” is the intercept constant; “β_i_”, “β_ii_”, and “β_ij_” are the regression coefficients of “F_1_”, “F_2_”, “F_3_”, “F_i_”, and “F_j_”, that are the coded values of antibiotics under examination; and “ε” is an error term.

Based upon this design (Table 3), the analysis of variance was performed which described the effect of all the selected antibiotics against bacterial isolates of bovine origin. The following fitted regression equation was obtained.

$$\mathrm{Inhibition}=7.00+0.06875A+0.3125B+1.00C+0.1875A^{2}+0.1875B^{2}-0.1875C^{2}-0.375\mathrm{AB}+0.00\mathrm{AC}+0.00\mathrm{BC}$$

**Figure 4:**
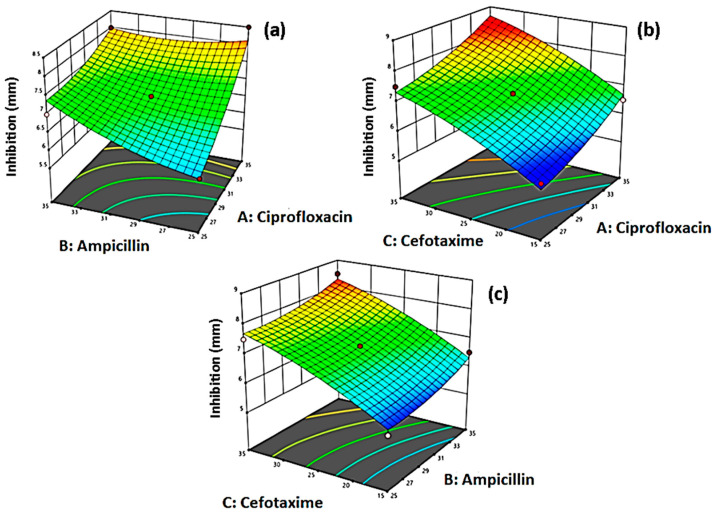
Response Surface plots for the inhibition of *C. perfringens* strains by (**a**) Ampicillin and Ciprofloxacin, (**b**) Ciprofloxacin and Cefotaxime, and (**c**) Ampicillin and Cefotaxime in livestock-producing areas of Punjab province in Pakistan. These plots are the depiction of response surface methodology by the Box–Behnken design.

Based on the analysis of variance (Table 4) applied to this model, there is a significant response at a 5% level of significance (*p* > 0.05), while this model shows very little lack of fit of data. The coefficient of determination (R^2^) also confirms that 96% of the variation in inhibition is accounted for by this model. Thus, the optimum dosage of the selected antibiotics is also defined in Table 5 and Figure 4 which determines maximum inhibition of bacterial isolates. Table 5 clarifies the concentrations of selected antibiotics in their combinations for substantial retardation of bacterial strains defined as inhibition (response variable). According to the values mentioned in Table 5, it has been concluded that the medium dose of antibiotics is optimum for substantial inhibition of bacterial strains. In run 16, three antibiotics at level 0 showed maximum inhibition of bacterium, while in run 10, ciprofloxacin and cefotaxime at a lower level, and ampicillin at medium level showed maximum inhibition. Similarly, in run 11, ciprofloxacin at a lower level, ampicillin at a high level, and cefotaxime at medium level showed maximum inhibition of bacterial strains.

Due to excessive application of antibiotics, the problem of antibiotic resistance is peaking towards a threatening situation, so to cope with this issue, the use of more than one antibiotic against a particular pathogen can be a useful strategy. To find the solution to the problem of antibiotic resistance, we checked those antibiotics which are in common use from a veterinary perspective. Eleven antibiotics were subjected to evaluation, and after careful examination, it was found that five antibiotics showed a significant inhibitory impact on bacterial isolates, while four antibiotics showed moderate inhibition, and two antibiotics showed no inhibition. Among these antibiotics, ciprofloxacin, ampicillin, and cefotaxime were found to be most effective. These results show some resemblance with Traub et al. (1986) in which they described that 106 isolates of *C. perfringens* were inhibited by ciprofloxacin, ampicillin, and cefotaxime from a total of 23 antimicrobial drugs [17]. While Kouassi et al. (2014) also confirmed that ciprofloxacin and cefotaxime were the most active antibiotics against *C. perfringens* in Cote d’Ivoire [18].

Determining the correlation of all antibiotics against the inhibition rate of bacterial isolates, it was observed that tetracycline, penicillin, erythromycin, and oxytetracycline showed less inhibition of isolates, while ciprofloxacin, ampicillin, and cefotaxime showed excellent/remarkable inhibitory response against the isolates. Lincomycin and amikacin showed no inhibition. Correlation also depicted the similarity patterns of the evaluated antibiotics against isolates. The conclusions drawn show some similarity with Kawamura-Sato et al. (2010) in which they observed that isolates of *Actinobacter* species were drastically inhibited with the aid of multiple antibiotics and their application at various times and doses [19].

Optimization of antibiotics application to bacterial isolates was performed under the Box–Behnken design (BBD) via response surface methodology (RSM), which confirms that ciprofloxacin and ampicillin showed maximum inhibition either applied as a single treatment or in combination, while cefotaxime used at lower concentration proved beneficial, and the isolated growth was very less which confirmed that judicious and appropriate application is helpful to combat the antibiotic resistance in bacteria. These results show resemblance with Shokoohi et al. (2018) in which they confirmed that antibiotics can also be checked against a particular pathogen using optimization via response surface methodology, and this method gave appreciable results [6]. El-Naggar et al. (2013) also described that Gram-positive and Gram-negative bacteria can be effectively inhibited by prevalent antibiotics; furthermore, the antibiotic resistance was greatly due to the application of multiple drugs available worldwide [3]. Furthermore, Anjum et al. (1997) and Liu et al. (1999) suggested that BBD is an efficient tool for determining the RSM for checking the optimization of various factors and conditions [14,18].

Optimization by RSM affects, by the selection of variable, which is the first and foremost step as the whole study is based on it. The appropriate selection of variables makes the experimental prediction easy and practically it becomes feasible [20]. Afterward, the choice of experimental design is crucial for the proper description and explanation of variables. The mathematical and statistical analysis to obtain the fitness of the data is necessary to attain an optimal region for all the treatments applied for obtaining an appropriate result [21].

The experimental variables and region should be properly defined as the number of variables that determine the order of the model that is either factorial or quadratic. In RSM, many designs are used to optimize the variables including Box–Behnken (BBD), Central Composite Design (CCD), and Doehlert designs [22]. The contour and surface plots developed after the analysis display optimal conditions of the applied treatments. These plots help in the visual inspection of the experiment. For quadratic models, the minimum–maximum and critical points are needed to determine the effectiveness of the mathematical function. This method is advantageous because it can properly describe a huge number of variables and their interactions [23]. So, our study illustrates that selection and usage of antibiotics conducted before RSM lead to appropriate forecasting of antibiotic combination.

This approach would help in minimizing the antibiotics resistance in the bovine population of Pakistan. Thus, the optimum therapeutic spectrum could be achieved by using three antibiotics, i.e., CAC, which could be a primary choice against *C. perfringens* infection in bovine species.

## 3. Materials and Methods

### 3.1. Sample Collection

**Figure 5:**
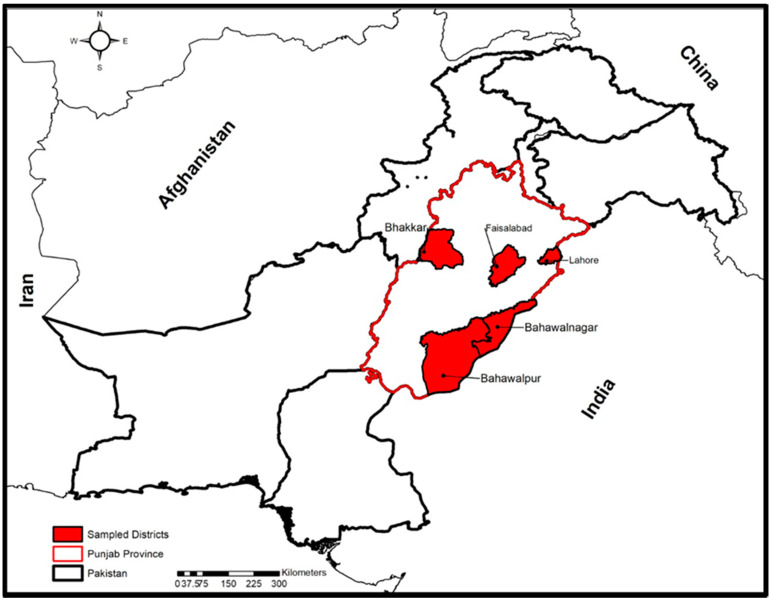
Map showing sampling sites for the collection of *C. perfringens* isolates in Punjab province of Pakistan. These sites include Lahore, Faisalabad, Bhakkar, Bahawalpur, and Bahawalnagar. White shaded portion with red boundaries depicts Punjab province and Red shaded portion with black boundaries are sites for sample collection.

This study encompassed the major livestock sites (Lahore, Faisalabad, Bhakkar, Bahawalpur, and Bahawalnagar) in the Punjab province of Pakistan (Figure 5). Intestinal contents of necropsied buffalo (*n* = 18) and cattle (*n* = 15) with a history of the intestinal problem were collected and transported to the laboratory for further processing.

### 3.2. Isolation of Bacterial Strains

Fecal swabs were inoculated into 5 mL thioglycolate (FTA) broth (Manufacturer: Huan Kai Microbial (HKM) Sci. & Tech, Guangzhou, China): for 1 L, 29.4 g of FTA medium was dissolved in 1000 mL of distilled water (as per the manufacturer’s recommendation) and incubated at 37 ℃ (Don Whitely DG-250 anaerobic workstation, United Kingdom) for 24 h. Subsequently, 100 μL of pre-enriched FTA broth was spread on tryptose sulphite cycloserine agar base enriched with 7% egg yolk and supplemented with D-cycloserine (Solarbio, Beijing, China). Multiple black colonies showing a positive lecithinase reaction were selected and cultured. For identification and purity of *C. perfringens*, they were streaked on Columbia blood agar (Huan Kai Microbial (HKM) Sci & Tech, Guangzhou, China) containing 5% defibrinated sheep blood and evaluated for typical double zone hemolysis associated with *C. perfringens*. Additionally, Gram staining and biochemical tests including glucose, maltose, H_2_S reduction test, nitrate reduction test, gelatin liquefaction, and saccharose test, etc. (Hangwei, Microbiological Co Ltd., Hangzhou, China), were performed to confirm the identity of the *C. perfringens*. Isolates were preserved in 50% glycerol at −80 °C till further use.

### 3.3. Antibiotic Susceptibility Testing

The isolated *C. perfringens* cultures were subjected to antibiotic sensitivity testing with selected antibiotics (Table 6 and Table 7) by using the antibiotics sensitivity discs of 9 mm (Oxoid™ Antimicrobial Susceptibility Disks Thermo Scientific™ USA) according to the standards procedures recommended by the Clinical Laboratory Standards Institute (CLSI) [24]. The antibiotic susceptibility test was performed according to the Kirby–Bauer method (i.e., Kirby–Bauer method is based on the inhibition of bacterial growth measured under standard conditions. For this test, a culture medium, mainly Mueller-Hinton agar, is uniformly and aseptically inoculated with the test organism, and then filter paper discs, which are impregnated with a specific concentration of a particular antibiotic, are placed on the medium. The organism will grow on the agar plate, while the antibiotic inhibits the growth. If the organism is susceptible to a specific antibiotic, there will be no growth around the disc containing the antibiotic. Thus, a zone of inhibition can be observed and measured to determine the susceptibility to an antibiotic for that particular organism). The antibiotics were evaluated for AMR (i.e., when microorganisms evolve mechanisms that protect them from the effects of antimicrobials. It applies to bacteria that become resistant to antibiotics) against 33 confirmed isolates of *C. perfringens* (*n* = 18 Buffalo; *n* = 15 Cow) based on the zone of inhibition (ZI). Antibiotic resistances were determined based on the criteria reported previously [2] as follows:

### 3.4. Statistical Analysis

The data were subjected to one-way analysis of variance (ANOVA), the means were compared using Tukey’s Honestly Significant Difference Test (HSD), and a Chi-square test was performed using the Statistical Package for Social Sciences (SPSS) software version 26.0 Armonk, NY, USA. Correlation of inhibition zone (IZ) values and cluster analysis amongst the antibiotics were performed using R Studio suite 1.3.1093 (package heatmaply and function heatmapr(), heatmaply()). Optimization of the parameters was conducted using response surface methodology (RSM) via the Box–Behnken design (BBD) on Design-Expert^®^ software version 12.0 (Design-Expert version 12 Stat-Ease Inc. Suite 6400, Minneapolis, MN 55413, USA)

## 4. Conclusions

The rationale of this study is to evaluate 11 common antibiotics against *C. perfringens*. Results depicted that CAC exhibited maximum inhibitory response, and were further subjected to optimization by RSM giving possible combinations of CAC that could substantially inhibit *C. perfringens* proliferation and resistance. Drug combinations could potentially be a better strategy for improving antimicrobial therapy. This methodology can be adopted in bovines to exploit evolutionary tradeoffs which could affect the rate of resistance evolution in predictable ways. Interdisciplinary research on drug combinations will lead to further advances in microbiology, evolutionary biology, systems biology, and allied fields.

## Figures and Tables

**Table 1:** Antibiotic resistance of 33 *C. perfringens* strains isolated from buffalo and cattle against 11 antibiotics at their standard concentration in the bovine population of Punjab province in Pakistan. The zone of inhibition were assessed based on the criteria reported by Hu et al. [2] as, “−” = diameter ≤ 5 mm (resistant), “+” = 5 mm < diameter ≤ 15 mm (less susceptible), “++” = 15 mm < diameter ≤ 25 mm (moderately susceptible) and “+++” = 25 mm < diameter ≤ 35 mm (highly susceptible).

	Isolates (*n* = 33)	Antibiotics and Their Concentrations										
		Penicillin (10 µg)	Vancomycin (5 µg)	Lincomycin (10 µg)	Amikacin (25 µg)	Oxytetracycline (30 µg)	Ampicillin (10 µg)	Cefotaxime (30 µg)	Tetracycline (30 µg)	Ciprofloxacin (30 µg)	Erythromycin (15 µg)	Metronidazole (5 µg)
Buffalo (*n* = 18)												
1	CP-LHE-B1-PK	++	+	−	−	+	+++	++	+	+++	+	++
2	CP-LHE-B2-PK	++	+	−	−	+	+++	++	+	+++	+	++
3	CP-LHE-B3-PK	++	+	−	−	+	+++	+++	+	+++	+	++
4	CP-PAT-B1-PK	+++	++	−	−	+	+++	+++	+	+++	+	+++
5	CP-PAT-B2-PK	+++	+	−	−	+	+++	++	+	++	+	++
6	CP-SGD-B1-PK	++	+	−	−	−	+++	+++	−	++	+	+++
7	CP-SGD-B2-PK	++	++	−	−	−	+++	+++	−	+++	+	++
8	CP-JHG-B1-PK	+++	+	−	−	−	++	++	−	+++	+	++
9	CP-JHG-B2-PK	+++	+	−	−	+	+++	+++	++	+++	+	++
10	CP-SHW-B1-PK	++	+	−	−	+	++	++	+	++	+	++
11	CP-SHW-B2-PK	+++	+	−	−	+	++	++	+	+++	+	++
12	CP-BWN-B1-PK	++	+	−	−	−	++	++	−	+++	+	++
13	CP-BWN-B2-PK	++	+	−	−	++	+++	++	++	+++	+	++
14	CP-BHK-B1-PK	++	+	−	−	+	+++	+++	+	+++	+	++
15	CP-BHK-B2-PK	++	+	−	−	+	+++	++	+	+++	+	++
16	CP-BHK-B3-PK	++	+	−	−	−	++	+++	−	+++	+	+++
17	CP-DGK-B1-PK	++	+	−	−	−	+++	++	−	+++	+	++
18	CP-DGK-B2-PK	++	+	−	−	−	++	+	+	+++	+	+++
Cattle (*n* = 15)												
1	CP-LHE-C1-PK	++	+	−	−	+	++	++	++	+++	+	++
2	CP-LHE-C2-PK	++	+	−	−	+	++	++	+	+++	+	++
3	CP-PAT-C1-PK	++	+	−	−	+	+++	+++	+	+++	+	++
4	CP-PAT-C2-PK	++	+	−	−	+	++	++	+	+++	+	++
5	CP-SGD-C1-PK	++	+	−	−	−	++	++	−	+++	+	++
6	CP-JHG-C1-PK	++	+	−	−	+	+++	+++	+	+++	+	+++
7	CP-SHW-C1-PK	++	++	−	−	−	++	++	−	+++	+	++
8	CP-SHW-C2-PK	++	+	−	−	−	+++	+++	−	+++	+	++
9	CP-BWN-C1-PK	++	+	−	−	+	+++	++	+	+++	−	+++
10	CP-BWN-C2-PK	+++	+	−	−	−	++	++	−	++	+	++
11	CP-BHK-C1-PK	+++	+	−	−	−	++	+	−	+++	+	++
12	CP-BHK-C2-PK	++	++	−	−	+	++	++	−	+++	+	+++
13	CP-BHK-C3-PK	+++	+	−	−	−	+++	++	−	++	−	+++
14	CP-DGK-C1-PK	++	+	−	−	+	+++	++	−	+++	+	++
15	CP-DGK-C2-PK	++	++	−	−	+	+++	++	+	+++	−	++

**Table 2:** Evaluation of inhibition activity of 11 antibiotics against *C. perfringens* isolates from various sites of Punjab province of Pakistan via Chi-square test; alphabetic letters represent the degree of inhibition activity.

Antibiotics	Chi-Square Value	*p*-Value
Penicillin	15.33 ^c^	0.003
Vancomycin	0.76 ^f^	0.000
Lincomycin	0.00	-
Amikacin	0.00	-
Oxytetracycline	8.91 ^d^	0.000
Ampicillin	16.55 ^b^	0.034
Cefotaxime	16.03 ^b^	0.000
Tetracycline	8.76 ^d^	0.012
Ciprofloxacin	22.09 ^a^	0.000
Erythromycin	7.76 ^e^	0.000

**Table 3:** A design approach for determining the optimization of antibiotics against bacterial inhibition in the Box–Behnken design.

Antibiotics	Coded Symbol	Range		
		−1	0	1
Ciprofloxacin	A	15	15	15
Ampicillin	B	25	25	25
Cefotaxime	C	35	35	35

**Table 4:** Analysis of variance of the selected antibiotics (Ciprofloxacin, Ampicillin, and Cefotaxime) for the response surface model against bacterial isolates activity via the Box–Behnken design.

Source	DF	SS	MS	F-Value	*p*-Value
Model	9	13.56	1.51	18.74	0.0004
A-Ciprofloxacin	1	3.78	3.78	47.06	0.0002
B-Ampicillin	1	0.7813	0.7813	9.72	0.0169
C-Cefotaxime	1	8.00	8.00	99.56	<0.0001
AB	1	0.5625	0.5625	7.00	0.0331
AC	1	0.0000	0.0000	0.0000	1.0000
BC	1	0.0000	0.0000	0.0000	1.0000
A^2^	1	0.1480	0.1480	1.84	0.0168
B^2^	1	0.1480	0.1480	1.84	0.0168
C^2^	1	0.1480	0.1480	1.84	0.0168
Residual	7	0.5625	0.0804		
Lack of Fit	3	0.5625	0.1875	2.33	0.961
Pure Error	4	0.0000	0.0000	-	-
Cor Total	16	14.12	-	-	-

R^2^ = 0.96.

**Table 5:** Observed and predicted values of selected antibiotics against bacterial isolates activity in the response surface model via the Box–Behnken design.

Runs	A Ciprofloxacin	B Ampicillin	C Cefotaxime	Inhibition of Strains (mm)	
				Observed	Predicted
1	0	−1	1	7.5	7.32
2	−1	0	1	5.5	5.21
3	0	−1	−1	6.00	5.78
4	0	0	0	7.00	6.72
5	0	1	−1	7.00	6.89
6	1	1	0	7.5	7.13
7	0	1	1	7.00	6.49
8	1	0	−1	7.00	6.31
9	0	0	1	5.5	5.11
10	−1	0	−1	8.5	8.41
11	−1	1	0	8.5	8.32
12	0	0	0	7.00	6.91
13	−1	−1	0	6.5	6.11
14	1	0	1	6.5	6.32
15	0	0	0	8.00	7.49
16	0	0	0	8.50	8.43
17	1	−1	0	7.00	6.91

**Table 6:** Physico-chemical properties of 11 antibiotics for the assessment of antimicrobial resistance against 33 isolates of *C. perfringens* isolated from livestock-producing sites in Punjab province of Pakistan.

Antibiotics	Class	Chemical Formula	Structural Formula	Molecular Weight (g mol^−1^)	Usage
Penicillin (Benzylpenicillin)	Penicillins	C_16_H_18_N_2_O_4_S	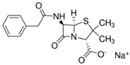	334.4	Bactericidal
Ampicillin	Penicillins	C_16_H_19_N_3_O_4_S	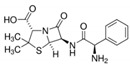	349.4	Bactericidal
Cefotaxime	Cephalosporins	C_16_H_17_N_5_O_7_S_2_	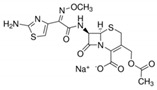	455.5	Bactericidal
Tetracycline	Tetracyclines	C_22_H_24_N_2_O_8_	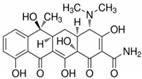	444.4	Bacteriostatic
Oxytetracycline	Tetracyclines	C_22_H_24_N_2_O_9_	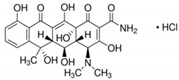	460.439	Bacteriostatic
Amikacin	Aminoglycosides	C_22_H_43_N_5_O_13_	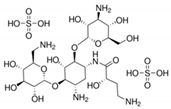	585.6	Bactericidal
Erythromycin	Macrolides	C_37_H_67_NO_13_	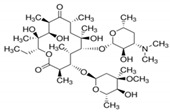	733.9	Bacteriostatic
Lincomycin	Lincosamide	C_18_H_34_N_2_O_6_S	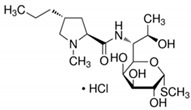	406.5	Bactericidal
Vancomycin	Glycopeptides	C_66_H_75_Cl_2_N_9_O_24_	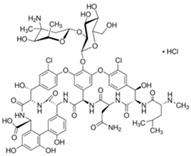	1449.2	Bactericidal
Ciprofloxacin	Fluoroquinolones	C_17_H_18_FN_3_O_3_	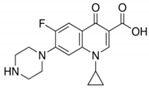	331.34	Bactericidal
Metronidazole	Nitroimidazoles.	C_6_H_9_N_3_O_3_	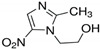	171.15	Bactericidal

**Table 7:** Antibiotic susceptibility testing scale based on zone of inhibition (mm).

Symbol	Zone of Inhibition (ZI)
−	diameter ≤ 5 mm
+	5 mm < diameter ≤ 15 mm
++	15 mm < diameter ≤ 25 mm
+++	25 mm < diameter ≤ 35 mm

## Data Availability

Not applicable.
